# Cytological markers used for identification and transfer of *Aegilops* spp. chromatin carrying valuable genes into cultivated forms of *Triticum*

**DOI:** 10.3897/CompCytogen.v13i1.30673

**Published:** 2019-02-27

**Authors:** Michał T. Kwiatek, Danuta Kurasiak-Popowska, Sylwia Mikołajczyk, Janetta Niemann, Agnieszka Tomkowiak, Dorota Weigt, Jerzy Nawracała

**Affiliations:** 1 Department of Genetics and Plant Breeding, Poznań University of Life Sciences, Dojazd 11, 60-632, Poznań, Poland Poznań University of Life Sciences Poznań Poland

**Keywords:** *
Aegilops
*, chromosome, banding, fluorescence *in situ* hybridization (FISH), genomic *in situ* hybridization (GISH), prebreeding, triticale, wheat

## Abstract

There are many reports describing chromosome structure, organization and evolution within goatgrasses (*Aegilops* spp.). Chromosome banding and fluorescence *in situ* hybridization techniques are main methods used to identify *Aegilops* Linnaeus, 1753 chromosomes. These data have essential value considering the close genetic and genomic relationship of goatgrasses with wheat (*Triticumaestivum* Linnaeus, 1753) and triticale (× *Triticosecale* Wittmack, 1899). A key question is whether those protocols are useful and effective for tracking *Aegilops* chromosomes or chromosome segments in genetic background of cultivated cereals. This article is a review of scientific reports describing chromosome identification methods, which were applied for development of prebreeding plant material and for transfer of desirable traits into *Triticum* Linnaeus, 1753 cultivated species. Moreover, this paper is a resume of the most efficient cytomolecular markers, which can be used to follow the introgression of *Aegilops* chromatin during the breeding process.

## Introduction

There are twenty three species of goatgrasses (*Aegilops* spp.) (Slageren 1994) and several of them are the closest relatives to wheats (*Triticum* spp.) (Kilian et al. 2011). The genomic constitution of goatgrasses is wide and include six genomes (D, S, U, C, N and M), which can be organized as diploids, tetraploids or hexaploids. What is more, most polyploid *Aegilops* Linnaeus, 1753 species are assumed to contain a common (pivotal) subgenome (U or D) while the second - differential genome (or genomes) is (are) much more genetically diversified (Zohary and Feldman 1962; Feldman and Levy 2012; Mirzaghaderi and Mason 2017). The evolution of *Aegilops* species was also intertwined with speciation of *Triticum* Linnaeus, 1753 forms (Goncharov 2011). It is reported that hexaploid wheat (*Triticumaestivum* Linnaeus, 1753; genomes AABBDD) originated through one or more hybridization events between a tetraploid wheat, *T.turgidum* Linnaeus, 1753 (genomes AABB), with the diploid goatgrass *Aegilopstauschii* Cosson, 1849 [genomes DD; syn. *Triticumtauschii* (Cosson,1849) Schmalhausen, 1897; syn. *Aegilopssquarrosa* auct. non Linnaeus, 1753, *Patropyrumtauschii* (Cosson, 1849) A. Love, 1984] (Kihara 1924, 1954; McFadden and Sears 1946). More precisely, Aegilopstauschiisubsp.strangulata (Eig, 1929) Tzvelev, 1973, has been accepted to be a donor of D-genome of wheat (Dvořák et al. 1998). Tetraploid wheat originated via hybridization of a species closely related to the extant *Aegilopsspeltoides* Tausch, 1837 [genomes SS; syn. *Sitopsisspeltoides* (Tausch, 1837) Á. Löve, 1984; syn. *Triticumspeltoides* (Tausch, 1837) Grenier, 1890], which contributed the wheat B genome (Sarkar and Stebbins 1956; Dvořák et al. 1993; Feldman and Levy 2012; Salse et al. 2008), with diploid wheat (genomes AA). The most likely donor of A-genome of polyploid wheats is *T.urartu* Tumanian ex Gandilyan, 1972 (Konarev et al. 1974; Petersen et al. 2006; Golovnina et al. 2009). Some reports describe both genera jointly, as *Aegilops-Triticum* complex (Li et al. 2015; Ozkan et al. 2003; Zohary and Feldman 1962). A close relationship between the genera *Aegilops* and *Triticum* is widely adopted for introducing new genes by interspecific hybridization into cultivated cereals (Ruban and Badaeva 2018). Such introgression forms are important genetic resources for breeding. These kinds of genetic stocks can be used as an interesting plant material to study the expression of alien traits and for mapping particular loci (genes) onto *Aegilops* chromosomes (Rakszegi et al. 2017).

The ability to distinguish alien chromosomes, which were introduced into a genetic background of an acceptor plant, is the initial step in characterization of introgression lines. The first chromosome identification studies in wheat were done by Sears (1948), who assigned the loci for several agronomic and morphological traits on particular chromosomes and chromosome arms. Later, in 1970s all chromosomes of wheat could be distinguished using the C-banding or N-banding techniques (Gill and Kimber 1974; Iordansky et al. 1978; Endo and Gill 1984; Lukaszewski and Xu 1995). In 1990s, molecular biology protocols were combined with classical cytogenetic techniques to develop the fluorescence *in situ* hybridization (FISH) method. FISH allows the identification of DNA sequences directly on the chromosomes.

The first molecular probes used for FISH purposes on *Aegilops*-*Triticum* chromosomes contained conserved high-copy sequences, such as telomere sequences or 5S and 45S ribosomal RNA genes (Gerlach and Bedbrook 1979; Gerlach and Dyer 1980). The number and distribution of rDNA loci mapped on chromosomes of species belonging to *Aegilops*-*Triticum* complex turned out to be invariant. Hence, these probes were often used as markers in evolution and speciation studies, as well as in the evaluation of interspecific divergence (Badaeva et al. 1996a; b; 2002; 2004; 2015). Mukai et al. (1993) used pSc119.2 and pAs1 sequences to identify all 21 chromosome pairs in wheat. Over time a number of cytomolecular markers were developed for the identification of chromosome arms or segments. For example, Cuadrado et al. (2000; 2008) used synthetic oligonucleotides (three base-pair repeats) to detect FISH signals on wheat chromosomes. BAC genomic libraries were also screened to develop FISH chromosome markers (Zhang et al. 2004). Komuro et al. (2013) screened 2000 plasmid wheat clones in order to detect multiple tandem repeated sequences, using *in situ* hybridization, and selected 47 of them, which gave clear signals on wheat chromosomes. Apart from physical mapping of DNA sequences onto chromosomes, the major breakthrough in chromosome identification was the development of an *in situ* hybridization technique utilizing total genomic DNA as a probe (GISH). This variant of *in situ* hybridization appeared to be a powerful tool for characterization of alien introgressions in cereals. The first GISH was carried out on chromosomes of synthetic hybrids of *Hordeumchilense* Roemer & Schultes, 1817 × *Secaleafricanum* Stapf, 1901 (Schwarzacher et al. 1989) and *Triticumaestivum* (wheat) × *S.cereale* Linnaeus, 1753 (rye) hybrids (Le et al. 1989). This technique is based on the divergence of repetitive DNA (Belyayev and Raskina 1998; Belyayev et al. 2001a; b) and was effectively used for identification of alien chromosomes/chromosome segments in hybrids or translocation lines of cereals (Schwarzacher et al. 1989; 1992; Leitch et al. 1990). GISH in combination with FISH was also used to study the genome constitution of natural and artificial hybrids, or to identify the introgression of alien chromosomes or chromosome segments (Jiang and Gill 2006).

The structure and organization of chromosomes of species belonging to the genera *Aegilops* and *Triticum* are collinear, as chromosomes within each homoeologous group are related by descent from a chromosome of the ancestor of the *Triticum*-*Aegilops* complex (Akhunov et al. 2003). Hence, large numbers of cytogenetic markers have a similar localization in the same homoeologous group (McCouch 2001). Moreover, this genetic resemblance can hamper the use of GISH in some instances (Majka et al. 2017). The synteny between the homoeologous *Aegilops* and *Triticum* chromosomes may be disturbed because of chromosome rearrangements, which appeared during the evolution process (Devos et al. 1993; Zhang et al. 1998). Moreover, it is known that the level of chromosome synteny decreases the more distant a chromosome region is from the centromere. It is also decreased in regions with increased meiotic recombination rates, also known as hotspots of recombination on chromosome arms (Akhunov et al. 2003). Such changes result in distribution variability of chromosome markers. This review summarizes cyto-molecular techniques, which differentiate *Aegilops* and *Triticum* chromosomes, and are used most often for effective tracking of *Aegilops* chromosomes (or chromosome segments) in cultivated cereals.

## Banding methods for identification of *Aegilops* chromatin introgression

Since the 1970s C-banding and N-banding techniques were used to distinguish the chromosomes of *Aegilops*-*Triticum* complex (Friebe et al. 1992; Gill and Kimber 1974; Landjeva and Ganeva 2000). C-banding has been employed to study genetic diversity and to create karyotypes of many *Aegilops* species. Giemsa C-banding was one of the first methods which allowed for identification of all 21 chromosome pairs of wheat (Endo 1986; Gill et al. 1991). This method was widely used to identify *Aegilops*-*Triticum* chromosome addition, substitution and translocation lines (Friebe et al. 1991; 1992; 1995; 1996a; 1996b; 1999; 2000; 2003). The results obtained by means of C-banding chromosome analysis of the majority of goatgrasses were reported in a series of articles describing the most important genomes of *Aegilops* (Badaeva et al. 1996a; 2002; 2004). C-banding analyses allowed the authors to discover that the S-genome of *Ae.speltoides* was most syntenic to B- and G-genomes of *Triticum*, but was different from other species of section Sitopsis (Badaeva et al. 1996a). Moreover, those authors observed minor polymorphisms in C-banding patterns of chromosomes of D-genome (Badaeva et al. 2002) and U-genome (Badaeva et al. 2004) belonging to different *Aegilops* species. All those results were later compared and confirmed by means of FISH studies (FISH methods are described in the third section of this review).

Polymorphisms in C-banding patterns were also utilised to distinguish *Aegilops* chromosomes in wheat genetic background. *Ae.speltoides* turned out to be one of the largest sources of valuable genes and was used to develop *Aegilops*-*Triticum* introgression lines. Friebe et al. (1991) used C-bands to establish the chromosome constitution of wheat streak mosaic virus (WSMV) and greenbug (*Schizaphisgraminum* Rondani, 1852) resistant lines, derived from wheat - *Agropyronintermedium* - *Aegilopsspeltoides* crosses. Three lines carried 7S(7A) chromosome substitution (derived from *Ae.speltoides*). The results indicated that the greenbug resistance gene *Gb5* was located on chromosome 7S. This chromosome was also used to transfer leaf rust (caused by *Pucciniatriticina* Eriksson, 1899) resistance gene combined with greenbug resistance gene *Gb5* into wheat genetic background (Dubcovsky et al. 1998). The authors induced a homologous recombination events using *ph1b* wheat mutant and developed Ti7AS-7S#1S-7AS.7AL translocation line conferring resistance to leaf rust and Ti7AS.7AL-7S#1L-7AL line conferring resistance to greenbug. The chromosome segments transferred from *Ae.speltoides* were characterized by means of C-banding and the fact of the translocation was supported by restriction fragment length polymorphisms (RFLP) analysis. Friebe et al. (1996a) applied C-banding analysis to identify T4AS.4AL-7S#2S chromosome translocations in wheat - *Ae.speltoides* lines with *Lr28* leaf rust resistance gene. Moreover, a chromosome translocation (2B.2S) involved in the *Lr35/Sr39* transfer derived from *Ae.speltoides* was identified using a C-banding method (Friebe et al. 1996a). C-banding technique was also used to determine the introgression carrying *Yr8/Sr34* yellow rust and stem rust resistance genes from *Ae.comosa* Smith, 1806 into wheat. Miller (1988) detected 2AS-2ML.2MS and 2DS-2ML.2MS chromosome translocations. Friebe et al. (1992) adopted the C-banding method and identified complete set of chromosomes of *Ae.caudata* Linnaeus, 1753 in the amphiploid *Triticumaestivum* cv ‘Alcedo’ - *Ae.caudata*. Furthermore, the authors developed six chromosome addition lines in which the *Ae.caudata* chromosome pairs were called B, C, D, F, E and G. Friebe et al. (1995) established a karyotype of *Ae.umbellulata* Zhukovsky, 1928 using C-banding analysis of ten accessions collected in ten different geographic locations. This approach allowed for the identification of individual alien chromosomes in wheat-*Ae.umbellulata* chromosome monosomic and telosomic addition and wheat - *Ae.umbellulata* translocation lines (Friebe et al. 1995).

One of the most notable applications of the C-banding technique was the identification of radiation-induced translocation lines resistant to leaf rust (*Lr9*) and assignment of *Lr9* loci to 6UL chromosome of *Ae.umbellulata*. The following chromosome translocations were identified by means of C-banding analysis: 6BL.6BS-6UL, T4BL.4BS-6UL, 2DS.2DL-6UL, T6BS.6BL-6UL and 7BL.7BS-6UL (Friebe et al. 1995). C-banding method was also used to identify 3BL.3BS-3S and 3DL.3DS-3S chromosome translocations conferring resistance to powdery mildew (*Pm13* gene), which was transferred from *Ae.longissima* Schweinfurth & Muschler, 1912 into wheat (Ceoloni et al. 1992; Friebe et al. 1996a). Another powdery mildew gene (*Pm32*) was transferred from *Ae.speltoides* into wheat and T1BL-1SS chromosomal translocation was revealed by means of C-banding analysis (Hsam et al. 2003). However, in some cases the C-banding method was not sufficient to discriminate between *Aegilops-Triticum* translocations. For example, C-banding patterns of the translocated 7DL arms from *Aegilopsventricosa* Tausch, 1837, carrying *Pch1* gene (responsible for resistance to eyespot) in cultivars Rendevous and Roazon was impossible to visualize as the patterns identified in 7DL chromosome of Chinese Spring wheat and 7DL of *Ae.ventricosa* were similar (Martin 1991). It was not until more sensitive C-banding protocol was applied that clear differences in the C-banding patterns between 7D of Chinese Spring and 7D of *Ae.ventricosa* were demonstrated by Badaeva et al. (2008). Another difficulty was reported by Apolinarska et al. (2010), who could not unambiguously identify the *Aegilopsvariabilis* Eig, 1929-rye chromosome translocations by means of C-banding.

The N-banding method was less often used to investigate *Aegilops-Triticum* introgression lines. Landjeva and Ganeva (1996; 2000) reported the N-banded karyotype of *Aegilopsovata* Linnaeus, 1753 (syn. *Ae.geniculata* Roth, 1787) and the chromosomal constitution of its partial amphiploid with bread wheat *Triticumaestivum* cv.‘Chinese Spring’. N-banding patterns made it possible to distinguish all *Ae.ovata* and wheat chromosomes. Ganeva et al. (2000) also used this technique, supported by gliadin electrophoresis, to reveal the structural changes in chromosomes 1A, 2A, 4B, 6B, 7B, 1D, and 2D of the *Ae.umbellulata*-wheat amphiploid (2n=6x=42, AABBUU), which showed leaf rust resistance conferred by *Lr9* gene homolog. C- and N-banding methods are effective techniques to distinguish alien chromatin in a large number of introgression lines. However, the precise identification of translocation breakpoints requires additional supporting technique – in most cases genomic *in situ* hybridization (GISH) would suffice.

## Fluorescence *in situ* hybridization methods for identification of *Aegilops* introgressions

A combination of molecular techniques and classical cytology became a breakthrough tool for science and crop breeding, especially for the development and characterization of *Aegilops-Triticum* introgression lines. First reports of adaptation of fluorescence *in situ* hybridization protocol for analyses of wheat chromosomes were published by Rayburn and Gill (1985) and Yamamoto and Mukai (1989). The ideal set of chromosome markers should cover the entire chromosome arms. This is a crucial requirement, which defines the usefulness of cytological landmarks for the identification of chromosome translocations. Hence, the most useful landmarks are DNA repetitive sequences that are richly represented in almost all chromosome regions, and can be used for evaluation of intra- and interspecific or intergeneric chromosome polymorphisms (Table 1).

**Table 1.** Tandem repeats used as effective FISH markers for identification of *Aegilops* chromatin introgression.

Tandem repeats	Clones/sequences	References
Satellite DNA sequences	pAs1, pSc119.2, pTa-71, pTa-86, pTa-465, pTa-535, pTa-566, pTa-713, pTa-794	Badaeva et al. 1996a; b; 2015; Schneider et al. 2005; Zhao et al. 2016; Kwiatek et al. 2015; 2016a; 2016b; 2017a; 2017b; Goriewa-Duba et al. 2018
Microsatellite DNA sequences (simple sequence repeats - SSR)	AAC, ACG, GAA	Molnar et al. 2005; 2011

To date the most popular probe used for identification of *Aegilops*-*Triticum* chromosomes is a D-genome specific repetitive DNA sequence called pAs1, derived from of *Aegilopssquarrosa* Linnaeus, 1753 (syn. *Ae.tauschii* Cosson, 1849; 2n = 14, genome DD) (Nagaki et al. 1995; Rayburn and Gill 1985). This sequence is AT rich (65.2%) and is widely distributed in many species of *Aegilops*-*Triticum* complex. It is included into *Afa-family* repeated sequences, because the recognition site of *AfaI* restriction enzyme was the most conserved sequence in this unit (Nagaki et al. 1995). Another much-used chromosome marker is a pSc119.2 repetitive sequence, derived from rye (*Secalecereale*) (Bedbrook et al. 1980). FISH landmarks of pSc119.2 and pAs1 are widely distributed in the chromosomes of *Aegilops* and *Triticum* species. A combination of those two probes was the first effective marker set used for chromosome identification of *Triticum* (Mukai et al. 1993) and *Aegilops* (Badaeva et al. 1996a; b; Schneider et al. 2005) species. However this set of markers was insufficient to describe some of *Aegilops* segments transferred into *Triticum* chromosomes. Hence, there was a need to develop more diversified and abundant chromosome landmarks.

**Figure 1.**
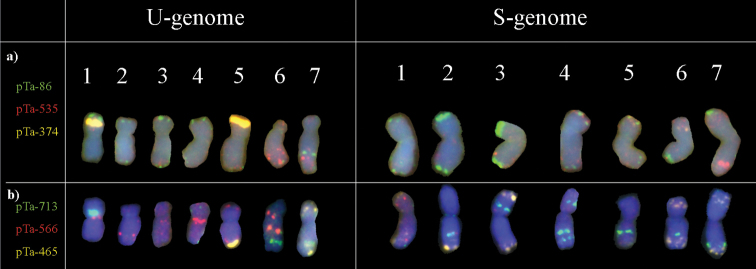
Karyograms of *Aegilopskotschyi* 2n=4x=28 chromosomes; UUSS) showing U- and S-genome chromosomes after two rounds of FISH with: **a** pTa-86 (green; Atto-488 fluorochrome; Jena Bioscience), pTa-535 (red; Atto-550 fluorochrome; Jena Bioscience), pTa-374 (25S rDNA; yellow; Atto-647 fluorochrome; Jena Bioscience) and **b** pTa-713 (green; Atto-488 fluorochrome; Jena Bioscience), pTa-k566 (red; Atto-550 fluorochrome; Jena Bioscience) and pTa-465 (yellow; Atto-647 fluorochrome; Jena Bioscience) probes (Kwiatek, unpublished)

Vershinin et al. (1994) identified dpTa1 family of repetitive sequences that are present in subtelomeric and interstitial regions of chromosomes belonging to *Aegilops*-*Triticum* complex. Salina et al. (2004; 1998; 2009) isolated, characterized and designated repetitive sequence called Spelt-1, which is located in subtelomeric regions of *Ae.speltoides*. Another repetitive sequence, Spelt52, pGC1R-1 belongs to the family of tandem repeats pAesKB52, located at subtelomeric regions of chromosomes *Ae.speltoides*, *Ae.longissima* and *Ae.sharonensis* Eig, 1928 (Anamthawat-Jonsson and Heslop-Harrison 1993; Zhang et al. 2002; Salina et al. 2004). Additionally, Kishii and Tsujimoto (2002) characterized TaiI family of tandem repeats, which are localized to the centromeric regions. Moreover, there are some groups of repetitive sequences, originated from related genera such as *Secale* sp. (subtelomeric repeats represented by 350 family pSc200 and pSc250) (Vershinin et al. 1994) and *Hordeumvulgare* Linnaeus, 1753 (HvRT telomere-associated sequences) (Kilian and Kleinhofs 1992), which are also represented in chromosomes of cultivated wheat or triticale. Other repetitive sequences that effectively discriminate between *Aegilops* and *Triticum* chromosomes were derived from BAC libraries of species belonging to Triticeae tribe. Komuro et al. (2013) screened 2000 plasmid wheat clones for signal occurence using FISH. 47 clones showed distinct signals on wheat chromosomes, and clones pTa-86 and pTa-535 were related to pSc119.2 and pAs1, respectively (Komuro et al. 2013). Kwiatek et al. (2017a; 2017b) used pTa-86, pTa-103, pTa-k374, pTa-465, pTa-535, pTa-k566, and pTa-713 to discriminate between the chromosomes of *Aegilopsbiuncialis* de Visiani, 1851, *Ae.ovata*, respectively and *Ae.kotschyi* Boissier, 1846 (unpublished, Figure 1) which were transferred into a triticale genetic background. This set of chromosome markers allowed for the identification of 1BS-1BL.5ML, 5MS-5ML.1BL, 7US.6BS-6BL, 6BS.7US-7UL, 1BS-1BL.5ML and 5MS-5ML.6BL chromosome translocations (Kwiatek et al. 2017a). Zhao et al. (2016) combined pSc119.2, pTa71 and pTa-713 and identified each of the 14 pairs of *Ae.variabilis* chromosomes.

Apart from the use of long repetitive sequences, one of the most effective ways to saturate chromosome regions with markers as much as possible is to apply microsatellite sequences as cytomolecular probes. Such trinucleotide sequences (i.e. AAC, GAA, ACG) were used to distinguish between chromosomes of wheat (Cuadrado et al. 2000) and *Aegilops* (Molnar et al. 2011). Furthermore, GISH effectively complemented FISH analysis so as to locate and identify the *Aegilops*-*Triticum* chromosome translocation breakpoints (Friebe et al. 1992; Kwiatek et al. 2017a). A combination of banding techniques and FISH/GISH methods were used for precise *Aegilops* chromosome identification in a *Triticum* background during the development of introgression lines with valuable traits. Friebe et al. (1995) combined C-banding and GISH using total genomic DNA of *Ae.umbellulata* to identify the chromosome breakpoints in radiation-induced *Triticum*-*Aegilops* translocation lines resistant to leaf rust (*Lr9*), which involved 4B and 6B chromosomes of wheat and 4U chromosome of *Ae.umbellulata*. In addition, Friebe et al. (2003) used C-banding and FISH to identify *Ae.sharonensis* chromosomes carrying gametocidal genes in a wheat genetic background. A 4BS.4BL-4S chromosome translocation was identified using clone pGclR-1, which is a 258 bp fragment of a tandem repetitive element and hybridizes to telomeric and subtelomeric regions of *Ae.speltoides*, *Ae.sharonensis*, and *Ae.longissima* chromosomes (Friebe et al. 2000).

A combination of C-banding and GISH methods was also used for development of wheat introgression lines with resistance genes against one of the most virulent races of stem rust (Pucciniagraminisvar.tritici Persoon, 1794), namely Ug99. Liu et al. (2011a) used this combination of cytomolecular methods, supported by SSR marker analysis, to identify three Robertsonian translocations (T3AL·3S^s^S, T3BL·3S^s^S and T3DL·3S^s^S) and one recombinant (T3DS-3S^s^S·3S^s^L) line with stem rust resistance as a common feature of the analysed forms. Faris et al. (2008) examined a durum wheat-*Aegilopsspeltoides* chromosome translocation line (DAS15), which was resistant to Ug99 and six other races of stem rust. GISH methods made it possible to identify 2BL-2SL.2SS translocation, which harbours stem rust resistance. GISH was also used to identify the 5DL-5M^g^L·5M^g^S chromosome translocation, which introduced resistance to stem rust races RKQQC and TTKSK (Ug99) into wheat (Liu et al. 2011b). Chromosome 5M^g^ of *Ae.geniculata* is also a source of leaf and yellow rust resistance genes (*Lr57* and *Yr40*, respectively). Kuraparthy et al. (2007) identified wheat-*Ae.geniculata* translocation lines (5DL·5DS-5M^g^S) using GISH. Molnar et al. (2005) combined GAA sequence probe with GISH to discriminate between the 1U, 2U, 4U and 5U chromosomes of *Ae.biuncialis* in wheat introgression lines, which showed limited tolerance to drought stress. Furthermore, Schneider et al. (2005) combined GISH and FISH using three repetitive DNA clones (pSc119.2, pAs1, and pTa71) to identify 2M, 3M, 7M, 3U, and 5U chromosome pairs in those lines. FISH/GISH methods, using pSc119.2, pAs1, 5S and 35S rDNA (from pTa71) sequence FISH probes together with GISH probes were also used to identify 2D^t^ and 3D^t^ chromosomes, carrying *Lr39* and *Lr32* genes, respectively in *Ae.tauschii*-triticale introgression lines (Kwiatek et al. 2015). The same set of FISH markers was used together with GISH to discriminate between 2S and 3S chromosomes of *Ae.variabilis*, which were transferred into triticale with intent to introduce the powdery mildew resistance gene *Pm13* (Kwiatek et al. 2016a). Mirzaghaderi et al. (2014) observed FISH patterns of the U^t^- and C^t^ -genome chromosomes of *Ae.triuncialis* Linnaeus, 1753 and *Ae.cylindrica* (Host, 1802) in wheat background. The following probes: pSc119.2-1, pTa535-1, pAs1-1, (CTT)_10_ and the 45S rDNA clone from wheat (pTa71), supported by GISH, were sufficient to discriminate between three different non-reciprocal homologous or heterologous translocations involving C^c^ and D^c^ chromosomes of *Ae.cylindrica*.

## Modifications and changes of FISH protocols for identification of *Aegilops* introgressions

In order to screen large populations of *Aegilops*-*Triticum* introgression forms, the methods for cytomolecular marker analysis should be easy to handle and cost-efficient. FISH protocols require fluorescent DNA probes, heat treatment and are labour and time consuming. There are reports describing modifications and changes to the protocols used to conduct repetitive sequence preparation for FISH. One of such techniques, primed *in situ* labeling (PRINS), combines polymerase chain reaction (PCR) with FISH to visualize sequences on chromosomes (Koch et al. 1989). This technique is based on the annealing of short, sequence-specific unlabelled DNA to denatured chromosomes (Kubalakova et al. 2001). Tang et al. (2014) designed oligonucleotides to replace the repetitive sequences pAs1, pSc119.2, pTa-535, pTa71, CCS1, and pAWRC.1 for *Aegilops-Triticum* chromosome identification. Kwiatek et al. (2016b) and Goriewa-Duba et al. (2018) developed specific primers to amplify some of the repetitive sequences reported by Komuro et al. (2013) from wheat genomic DNA. This approach reduces the time and the costs of BAC library maintenance. The modifications of FISH protocols also facilitate the chromosome identification. Cuadrado and Jouve (2010) investigated telomeres of barley (*Hordeumvulgare* L.) using non-denaturing FISH (ND-FISH). This method was used to study chromosomes of *Triticum* (Fu et al. 2015). The analytical potential of this technique was demonstrated by Tang et al. (2018), who developed new oligo probes that make possible the identification of particular chromosomal segments, i.e.: the intercalary regions of 4AL and 2DL chromosome arms, and the pericentromeric regions of 3DL and 6DS arms of wheat chromosomes.

Another way to saturate the chromosome arms with markers is the use of cDNA probes. Danilova et al. (2014) carried out FISH experiment with more than 60 full length wheat cDNAs, which were selected using BLAST against mapped EST markers (expressed sequence tags). FISH analysis revealed 1U-6U chromosome translocation in *Aegilopsumbellulata* and showed synteny between chromosome A of *Ae.caudata* and group-1 wheat chromosomes. There are certain reports, showing technical modifications of FISH procedures, which reduce the time and costs of experiments. For example, Kwiatek et al. (2016b) used four different fluorescence labels (Atto488, Atto550, Atto647 and DAPI) that made possible the examination of three different probes at the same time. Of course, this approach requires investing in excitation wavelength specific filter cubes, which are cost-consuming. When there is a need to examine hundreds of plants resulting from genetic crosses, in some cases the time and labour consuming cytological methods could be substituted. For example, Rey and Prieto (2017) used dot-blot genomic hybridization experiments instead of microscopy to detect alien genetic introgressions to bread wheat.

## Closing remarks: large scale selection of *Aegilops-Triticum* introgressions, perspectives for the future

Cytogenetic methods seem to be essential to verify genomic constitution in interspecific hybrids. The main problems are: limited sensitivity and spatial resolution, laborious and expensive protocols, which seriously limit the application of cytogenetic markers for large scale selection of *Aegilops*-*Triticum* introgressions. High-resolution and high-throughput methods are being progressively developed for identification of micro-introgressions, chromosome breakpoints and spatial localization of alien chromatin in donor nuclei. These require the use of new DNA markers, sequencing and new combinations of cytomolecular techniques. For example, three dimension FISH (3D-FISH) was applied to track the spatial organization of rye chromatin in wheat host genome (Burešová 2018). However, the main aim for development of *Aegilops*-*Triticum* introgressions is the transfer of desirable genes. Hence, there is a need to improve the cytogenetic methods for single gene physical mapping. Danilova et al. (2014) used single copy gene FISH with probes developed from cDNA of cytosolic acetyl-CoA carboxylase (ACCase) gene (Acc-2) and mapped them onto chromosomes of wheat. Another promising tool can be the combination of CRISPR (clustered regularly interspaced short palindromic repeats) with FISH. Deng et al. (2015) used a bacterial protein, CRISPR, combined with RNA sequences as probes to find the genes of interest. This method is comparably rapid and allows for keeping natural organization of the nucleus. What is more, CRISPR-FISH enables the analysis of spatial relationships between the genetic elements that are significant for gene expression. Apart from identification of *Aegilops*-*Triticum* introgressions, newly developed cytogenetic markers and methods could shed some light on the behaviour of chromatin, incorporated into the wheat genome, and show the results of the interaction between wheat genome and expression of introduced alien genes.
